# Median Arcuate Ligament Syndrome Beyond the Classic Demographic: Celiac Artery Compression in a 62-Year-Old Man With Cardiovascular Risk Factors

**DOI:** 10.7759/cureus.111180

**Published:** 2026-06-19

**Authors:** Rohan Kubba, Lucas Owens, Maani Bahador, Gregory Robinson, Nalin Ranasinghe, Leonard Ranasinghe

**Affiliations:** 1 Medicine, California Northstate University College of Medicine, Elk Grove, USA; 2 Clinical Medicine, California Northstate University College of Medicine, Elk Grove, USA

**Keywords:** atherosclerosis, atypical mals, celiac artery compression, median arcuate ligament syndrome, minimally invasive decompression, postprandial epigastric pain

## Abstract

Median arcuate ligament syndrome (MALS) is an uncommon vascular condition caused by external compression of the celiac artery by the median arcuate ligament. Although it predominantly affects young women, late-onset presentations in older men have been reported and may reflect a different underlying mechanism. We present a 62-year-old man with dyslipidemia and a 20-pack-year smoking history who came to the emergency department with chronic postprandial epigastric pain. Physical examination was unremarkable. There was no abdominal bruit and no tenderness to palpation. Routine blood work showed a mildly elevated prothrombin time (14.4 seconds; international normalized ratio (INR): 1.30), an intermediate Fibrosis-4 (FIB-4) score of 2.39, and an elevated mean platelet volume (MPV) (10.5 fL). Contrast-enhanced CT of the abdomen and pelvis at Bayonne Medical Center demonstrated moderate to severe stenosis of the celiac axis origin related to acute angulation, with post-stenotic dilatation, and findings correlating with median arcuate ligament syndrome per the final radiology report. No other cause of his symptoms was identified. He was transferred to a tertiary care facility and underwent a minimally invasive median arcuate ligament decompression without complications. This case raises awareness of MALS as a diagnosis that should be considered in older male patients with cardiovascular risk factors and unexplained postprandial pain. The severity of stenosis in this case, beyond what is typically seen in young women with classic MALS, suggests that intrinsic atherosclerotic disease may compound extrinsic ligamentous compression in older patients.

## Introduction

Median arcuate ligament syndrome (MALS), sometimes called celiac axis compression syndrome or Dunbar syndrome, occurs when the median arcuate ligament puts pressure on the origin of the celiac artery [1]. Classically, during expiration, the celiac trunk is at its highest level of stenosis, and on inspiration, luminal narrowing is typically decreased due to the action of the diaphragm. The median arcuate ligament has a fibrous structure. It is formed by the joining of the right and left diaphragmatic crura and normally sits above the celiac trunk. In roughly 10%-24% of people, it crosses lower than usual and contacts the artery, although only about 2 per 100,000 individuals ever develop symptoms [2]. The condition has long been associated with young, thin women between ages 20 and 45, and women are affected 3-4 times more often than men [3].

Symptoms are thought to arise through two intertwined pathways: reduced blood flow to the foregut from the compressed artery and pain from irritation of the surrounding celiac plexus [4]. Patients typically report cramping epigastric pain that comes on after meals, often accompanied by nausea and unintentional weight loss. Because these complaints mimic a long list of more common diagnoses, including peptic ulcer disease, pancreatitis, and mesenteric ischemia, MALS is almost always a diagnosis of exclusion. Cross-sectional imaging showing narrowing of the celiac artery at its origin is what ultimately confirms it. The characteristic imaging findings include the hook sign, a downward deflection of the proximal celiac trunk on sagittal views, and post-stenotic dilatation distal to the point of compression [2]. The combination of these features helps distinguish hemodynamically significant stenosis from incidental anatomic contact between the ligament and the vessel.

Most cases are thought to arise from a structural abnormality present from birth. However, a growing body of case reports describes MALS in older patients, with proposed mechanisms including caudal shifts in spinal curvature with aging, significant weight loss causing descent of intra-abdominal structures, and atherosclerotic remodeling of the vessel wall that may narrow an already compressed lumen [5]. Collectively, these reports suggest that anatomic compression previously subclinical throughout adulthood can become hemodynamically significant later in life, particularly when compounded by intrinsic vascular changes.

## Case presentation

A 62-year-old man with a history of dyslipidemia and a 20-pack-year smoking history came to the emergency department at Bayonne Medical Center, reporting chronic, dull epigastric pain that was evident after eating and had progressively worsened over several months prior to presentation. In the weeks preceding presentation, the patient experienced a notable escalation in his baseline pain that prompted him to seek emergency evaluation. He had no prior abdominal surgeries and no history of gastrointestinal bleeding or malignancy. He denied nausea, vomiting, changes in bowel habits, or weight loss. The patient did not report taking any prescription medications or anticoagulants at home.

Physical examination

At presentation, the patient was alert and oriented to person, place, and time, with normal concentration and insight. His temperature was 98.3°F, heart rate 94 beats per minute, blood pressure 136/72 mmHg, respiratory rate 20 breaths per minute, and oxygen saturation 98% on room air. He appeared thin. Heart sounds were normal, and peripheral pulses were strong and symmetric. The abdomen was soft and nontender throughout, with no guarding, no rebound, and no epigastric bruit on auscultation. Bowel sounds were present and active. Strength and sensation were intact in all extremities, and the spine was nontender throughout.

Laboratory findings

Complete blood count with differential showed a white blood cell count (WBC) of 4.4 x 10^9^/L, hemoglobin (Hgb) of 14.2 g/dL, and platelet count of 165 x 10^9^/L. The mean platelet volume (MPV) was elevated at 10.5 fL. The differential was notable for a low neutrophil percentage (42.2%) and elevated monocyte (12.7%) and basophil (1.8%) percentages. The comprehensive metabolic panel showed a mildly elevated blood urea nitrogen (BUN) of 22 mg/dL with a creatinine of 1.0 mg/dL and an estimated glomerular filtration rate (eGFR) of 85 mL/min/1.73 m². Liver enzymes were normal: aspartate aminotransferase (AST), 27 U/L; alanine aminotransferase (ALT), 18 U/L; alkaline phosphatase, 55 U/L; total bilirubin, 0.6 mg/dL; total protein, 8.1 g/dL; and albumin, 4.7 g/dL. Amylase was within reference range at 93 U/L, while lipase was borderline elevated at 65 U/L. Troponin I was negative. Lactic acid drawn on a subsequent sample was 0.6 mmol/L.

Coagulation testing showed a prothrombin time of 14.4 seconds (reference range: 11.0-13.5 seconds) with an international normalized ratio (INR) of 1.30 (reference range: 0.8-1.1 in patients not on anticoagulation) and an activated partial thromboplastin time of 31.9 seconds. Based on the parameters proposed by Sterling et al., the patient had a Fibrosis-4 (FIB-4) score of 2.39, which placed him in the intermediate-risk zone for significant hepatic fibrosis [6]. Urinalysis was unremarkable.

A summary of all laboratory values is provided in Table 1.

**Table 1:** Cumulative laboratory values Units: mg/dL: milligrams per deciliter, mEq/L: milliequivalents per liter, U/L: units per liter, ng/mL: nanograms per milliliter, mmol/L: millimoles per liter, /hpf: per high‑power field Abbreviations: CBC: complete blood count, WBC: white blood cell count, RBC: red blood cell count, Hgb: hemoglobin, Hct: hematocrit, MCV: mean corpuscular volume, MCH: mean corpuscular hemoglobin, MCHC: mean corpuscular hemoglobin concentration, RDW: red cell distribution width, MPV: mean platelet volume, CO2: carbon dioxide, BUN: blood urea nitrogen, eGFR: estimated glomerular filtration rate, AST: aspartate aminotransferase, ALT/SGPT: alanine aminotransferase/serum glutamic-pyruvic transaminase, FIB-4: Fibrosis-4, LDH: lactate dehydrogenase, CK: creatine kinase, INR: international normalized ratio

CBC with differential (day 1)				
Test	Result	Reference range	Units	Interpretation
WBC	4.4	4.5-11.0	×10³/µL	Borderline low
RBC	4.63	4.50-5.90	×10⁶/µL	Within reference range
Hgb	14.2	13.5-17.5	g/dL	Within reference range
Hct	41.5	41.0-53.0	%	Within reference range
MCV	89.7	80.0-100.0	fL	Within reference range
MCH	30.6	27.0-33.0	pg	Within reference range
MCHC	34.2	32.0-36.0	g/dL	Within reference range
RDW	13.7	11.5-14.5	%	Within reference range
Platelet count	165	150-400	×10³/µL	Within reference range
MPV	10.5	7.5-10.0	fL	Borderline elevated
Neutrophils %	42.2	50-70	%	Low
Lymphocytes %	41.3	20-40	%	Borderline elevated
Monocytes %	12.7	2-10	%	Elevated
Eosinophils %	2.0	1-4	%	Within reference range
Basophils %	1.8	0-1	%	Elevated
Lymphocytes # (Abs)	1.8	1.0-4.0	×10³/µL	Within reference range
Monocytes # (Abs)	0.6	0.2-0.8	×10³/µL	Within reference range
Eosinophils # (Abs)	0.1	0.0-0.4	×10³/µL	Within reference range
Basophils # (Abs)	0.1	0.0-0.1	×10³/µL	Within reference range
Absolute neutrophils	1.9	1.8-7.7	×10³/µL	Within reference range
Monocyte distribution width	15.50	12.0-17.5	-	Within reference range
Comprehensive metabolic panel (day 1)				
Test	Result	Reference range	Units	Interpretation
Sodium	138	136-145	mEq/L	Within reference range
Potassium	4.1	3.5-5.1	mEq/L	Within reference range
Chloride	106	98-107	mEq/L	Within reference range
CO2	25	22-29	mEq/L	Within reference range
Anion gap	10	8-16	mEq/L	Within reference range
BUN	22	7-20	mg/dL	Borderline elevated
Creatinine	1.0	0.7-1.2	mg/dL	Within reference range
eGFR (CKD-EPI)	85	≥60	mL/min/1.73m²	Within reference range
Random glucose	95	70-140	mg/dL	Within reference range
Calcium	9.8	8.5-10.5	mg/dL	Within reference range
Total bilirubin	0.6	0.1-1.2	mg/dL	Within reference range
AST	27	10-40	U/L	Within reference range
ALT/SGPT	18	7-56	U/L	Within reference range
Alkaline phosphatase	55	44-147	U/L	Within reference range
FIB-4 score	2.39	<1.30 low risk/>2.67 high risk	-	Indeterminate (1.30-2.67)
LDH	159	122-222	U/L	Within reference range
Total protein	8.1	6.3-8.2	g/dL	Within reference range
Albumin	4.7	3.5-5.0	g/dL	Within reference range
Globulin	3.4	2.0-3.5	g/dL	Within reference range
Albumin/globulin ratio	1.4	1.0-2.5	-	Within reference range
Amylase	93	30-110	U/L	Within reference range
Lipase	65	13-60	U/L	Borderline elevated
Cardiac markers and coagulation (day 1) (lactic acid (day 2))				
Test	Result	Reference range	Units	Interpretation
Troponin I	<0.01	<0.04	ng/mL	Within reference range
Total CK	165	39-308	U/L	Within reference range
INR	1.30	0.80-1.20	-	Borderline elevated
Lactic acid	0.6	0.5-2.2	mmol/L	Within reference range (day 2)
Urinalysis (day 1)				
Test	Result	Reference range	Units	Interpretation
Urine color	Light yellow	Yellow	-	Within reference range
Urine clarity	Clear	Clear	-	Within reference range
Urine pH	5.0	4.5-8.0	-	Within reference range
Specific gravity	1.022	1.001-1.035	-	Within reference range
Urine protein	Negative	Negative	-	Within reference range
Urine glucose	Normal	Negative	-	Within reference range
Urine ketones	Negative	Negative	-	Within reference range
Urine blood	Negative	Negative	-	Within reference range
Urine nitrite	Negative	Negative	-	Within reference range
Urine bilirubin	Negative	Negative	-	Within reference range
Urine urobilinogen	Normal	Normal	mg/dL	Within reference range
Leukocyte esterase	Negative	Negative	-	Within reference range
Urine WBC (auto)	<1	<5	/hpf	Within reference range
Urine RBC (auto)	<1	<3	/hpf	Within reference range
Squamous epithelial cells	<1	<5	/hpf	Within reference range

Imaging

CT of the abdomen and pelvis with intravenous contrast was performed at Bayonne Medical Center on day 2. Images were reconstructed in the axial plane and reformatted in sagittal and coronal views. The liver, gallbladder, bile ducts, pancreas, spleen, adrenal glands, kidneys, ureters, stomach, and bowel were all unremarkable. The appendix was normal. No free air, ascites, bowel obstruction, adenopathy, or acute fracture was identified. An incidental retroaortic left renal vein was noted.

The vascular findings were significant. The final radiology report on day 2 read: “Incidental moderate to severe stenosis of celiac axis origin, which may be related to median arcuate ligament due to acute angulation. Recommend clinical correlation for median arcuate ligament syndrome.” This represented an upgrade from the preliminary overnight interpretation, which had described only mild post-stenotic dilatation and pointed to a hemodynamically more significant degree of compression.

Annotated sagittal and coronal CT images are shown in Figure 1 and Figure 2. The sagittal view demonstrates the characteristic hook sign at the celiac trunk origin, with the vessel deflected sharply downward at the point of compression. Arrows and a circle mark the stenotic and post-stenotic segments. Given the clinical picture and the absence of any other explanation on laboratory or imaging workup, a diagnosis of MALS was made.

**Figure 1:**
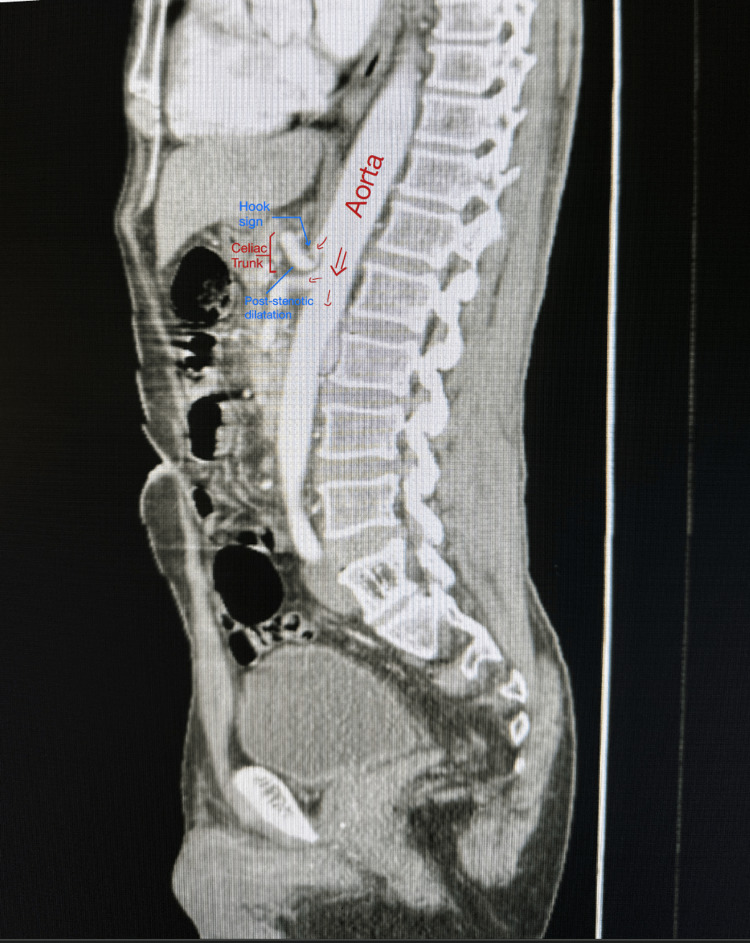
Sagittal CT The aorta (labeled in red) serves as the primary anatomic landmark and is visible as the large midline vessel anterior to the lumbar spine. The celiac trunk (labeled in blue) arises from the anterior aortic wall and demonstrates focal narrowing at its origin due to extrinsic compression by the median arcuate ligament. The hook sign (labeled in blue) refers to the characteristic downward deflection of the proximal celiac trunk at the site of maximal compression, produced by the low-lying fibrous arch of the median arcuate ligament crossing the vessel anteriorly. Red arrows identify the site of stenosis. Post-stenotic dilatation (labeled in blue) is visible distal to the point of compression and confirms the hemodynamic significance of the stenosis.

**Figure 2:**
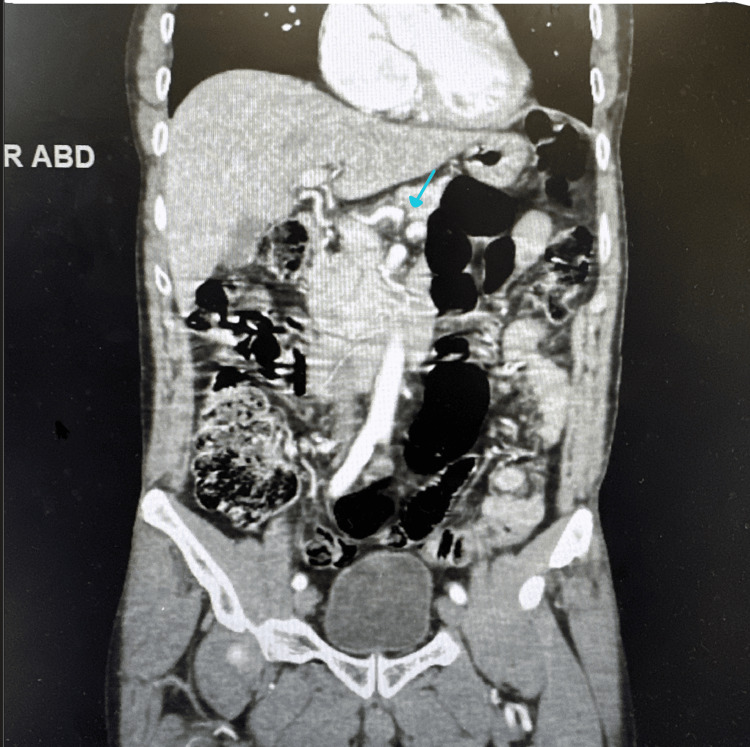
Coronal contrast-enhanced CT The blue arrow demonstrates the trifurcation of the celiac trunk into its major branches. This view provides complementary anatomical context for the vascular relationships identified on sagittal imaging and is consistent with the celiac artery origin narrowing described in the formal radiology report.

Management

During his emergency department stay, the patient was treated with intravenous fluids, intravenous pantoprazole, and intravenous acetaminophen. The on-call surgical team was consulted, and the case was discussed with vascular surgery. Given the degree of stenosis and the clinical findings, the decision was made to transfer him to a tertiary facility for formal vascular evaluation. He subsequently underwent a minimally invasive median arcuate ligament decompression procedure without perioperative complications. Long-term follow-up regarding symptom resolution was unavailable due to his transfer to an outside tertiary care system, which is a noted limitation to this case report.

## Discussion

This case stands out not simply because an older man was found to have MALS, but rather because of the degree of stenosis identified on imaging and what that may suggest about how the disease develops outside the classic demographic. The final radiology report described moderate to severe stenosis at the celiac origin, a severity beyond the mild to moderate narrowing commonly reported in younger women with classic MALS, where even symptomatic cases often show less dramatic stenosis [2]. In those typical presentations, the presence of post-stenotic dilatation serves as a critical radiologic marker confirming that the stenosis is hemodynamically significant and not simply an incidental anatomic variant. Post-stenotic dilatation develops when a pressure gradient across a stenotic segment produces turbulent downstream flow, and it is present in the majority of symptomatic patients with MALS regardless of age or sex [2]. Its presence helps distinguish true disease from the much larger population of individuals who have incidental median arcuate ligament contact with the celiac artery on imaging but no symptoms.

In this patient, however, the stenosis was graded as moderate to severe rather than mild, which is significant. The presence of dyslipidemia and a 20-pack-year smoking history raises the possibility that atherosclerotic plaque at the celiac ostium contributed to a functionally tighter stenosis than the ligamentous compression alone would have produced [7]. It must be emphasized, however, that this remains speculative: no gross calcifications were identified on CT, no angiographic data were obtained, and no pathologic confirmation of atherosclerotic involvement was available. The post-stenotic dilatation observed here is therefore most directly attributable to extrinsic ligamentous compression, with intrinsic atherosclerotic disease representing a plausible but unconfirmed contributing mechanism. Compression of the celiac nerve plexus is likely what prompts symptomatic MALS, and surgeons will typically consider removing portions of this during surgery to potentially decrease chronic neuropathic pain.

Dung et al. described a similar scenario in 2025, reporting a 66-year-old woman in whom MALS coexisted with atheromatous changes at the celiac trunk, producing a dual mechanism of obstruction [7]. The present case adds a male patient to that picture, with a more severe radiographic degree of stenosis and a clinical presentation that brought him to an emergency department rather than a scheduled outpatient workup. The pattern across these cases suggests that cardiovascular risk factors may amplify the hemodynamic consequences of anatomic compression, particularly in patients who are past middle age and in whom plaque burden at major branch points is more likely.

The physical examination findings here are worth addressing directly. No epigastric bruit was heard, and there was no tenderness to palpation. Both of these are often cited as hallmarks of MALS, but neither is reliably present. Bruits reflect turbulent flow and are absent in many confirmed cases, particularly when the stenosis is severe enough to reduce flow significantly or when body habitus limits transmission [8]. Absence of tenderness is similarly common in MALS because the pain mechanism is ischemic and neuropathic rather than inflammatory, and examination findings tend to be out of proportion to the severity of the underlying problem [4].

Relatively few laboratory values in this case were abnormal. The prothrombin time of 14.4 seconds with an INR of 1.30 is mildly elevated for a patient not on anticoagulation. Mild prolongation of coagulation studies can reflect subtle impairment of hepatic synthetic function, particularly given that the hepatic artery arises from the celiac axis and may be affected by chronic reductions in celiac perfusion. The patient’s Fibrosis‑4 (FIB‑4) score of 2.39 falls in the intermediate range for hepatic fibrosis risk, which warrants longitudinal follow‑up but does not, by itself, establish clinically significant liver disease. Importantly, his liver enzymes and albumin were within reference range. While the patient did not report taking home anticoagulants, the subtle isolated INR elevation is likely multifactorial, and attributing it solely to celiac hypoperfusion remains speculative. These findings are noted as observations that may merit future prospective study rather than conclusions supported by this single case.

The low-normal lactic acid level of 0.6 mmol/L is also informative. It effectively rules out acute gut ischemia and supports the interpretation that collateral perfusion from the superior and inferior mesenteric arteries was compensating adequately despite the celiac stenosis [8]. This is consistent with the chronic, compensated nature of ligamentous compression rather than an acute occlusive event.

From a systems perspective, this case illustrates a pattern that is easy to miss. An older man with vague postprandial pain and a largely unremarkable examination is far more likely to leave an emergency department with a diagnosis of dyspepsia, gastritis, or functional abdominal pain than with a confirmed vascular diagnosis requiring transfer to a tertiary center. The fact that the CT was performed and interpreted carefully, with the radiologist specifically flagging the celiac finding for clinical correlation, was the critical step. Standard contrast-enhanced CT is sufficient to make this diagnosis when the imaging is read carefully, and this case reinforces that emergency providers should not wait for dedicated vascular imaging when the clinical picture and CT findings are consistent [2,6].

Several limitations of this case should be acknowledged. First, long-term postoperative follow-up was unavailable because the patient was transferred to an outside tertiary care system; symptom resolution following decompression, which is an important criterion supporting the diagnosis of symptomatic MALS, could therefore not be confirmed. Second, while the patient did not report taking home anticoagulants, the possibility of an unrecognized over-the-counter supplement or drug-related contribution to the mildly elevated INR cannot be entirely excluded. Third, the hypothesis that atherosclerotic disease compounded extrinsic celiac compression is not supported by direct radiologic, angiographic, or pathologic evidence and remains speculative. These limitations are inherent to the retrospective, single-center nature of a case report and should be considered when interpreting the findings.

## Conclusions

This case adds to the evidence that MALS is not exclusively a diagnosis of young women and that older men with atherosclerotic risk factors can develop clinically significant celiac artery compression. The degree of stenosis seen here, characterized as moderate to severe on the final radiology read, is consistent with hemodynamically significant extrinsic ligamentous compression. Whether intrinsic atherosclerotic disease contributed to the severity of stenosis cannot be confirmed from the available data and should be regarded as a hypothesis rather than a conclusion.

While post-stenotic dilatation is a common finding in symptomatic patients with MALS across all demographics, the severity of stenosis in this case exceeds what is typically seen in younger patients with purely anatomic compression. Recognizing this compounded mechanism is important because it broadens the demographic for which MALS should be considered and suggests that cardiovascular risk factors may be a useful clinical cue when evaluating older patients with unexplained postprandial epigastric pain. Prospectively, the mildly elevated INR and intermediate FIB‑4 score may suggest that chronic celiac hypoperfusion could begin to affect hepatic synthetic function, a potential association that is not well studied and merits further evaluation in future prospective work. Providers should be willing to consider MALS when imaging flags a celiac anomaly, even in patients who do not match the classic profile, because early recognition and surgical referral remain the most effective path to symptom resolution.
